# Educational technologies for accident prevention due to falls in childhood: a scoping review

**DOI:** 10.1590/0034-7167-2022-0807

**Published:** 2023-12-08

**Authors:** Abiúde Nadabe e Silva, Aline Costa de Oliveira, Jefferson Abraão Caetano Lira, Ana Roberta Vilarouca da Silva, Lídya Tolstenko Nogueira

**Affiliations:** I Universidade Federal do Piauí. Teresina, Piauí, Brazil

**Keywords:** Child, Child, Preschool, Educational Technology, Accident Prevention, Accidental Falls, Niño, Preescolar, Tecnología Educacional, Prevención de Accidentes, Accidentes por Caídas, Criança, Pré-Escolar, Tecnologia Educacional, Prevenção de Acidentes, Acidentes por Quedas

## Abstract

**Objectives:**

to map evidence on educational technology use for accident prevention due to falls in childhood.

**Methods:**

a scoping review, carried out in October and November 2022, in the MEDLINE, Web of Science, BDENF and CINAHL databases and LILACS bibliographic index. There was no delimitation of language or time. Data were extracted and analyzed descriptively by two independent researchers. The research protocol was registered in the Open Science Framework.

**Results:**

twenty-six studies were selected. Booklets, pamphlets and leaflets were the most used technologies, presenting health services as the most frequent environment to develop research on fall prevention. The technologies developed were important outcomes: increased knowledge of children, family members, caregivers, health and education professionals.

**Conclusions:**

educational technology use makes it possible to increase knowledge, adopt safe practices and reduce falls.

## RESUMO

**Objetivos:**

mapear evidências sobre a utilização de tecnologias educacionais para a prevenção de acidentes por quedas na infância.

**Métodos:**

revisão de escopo, realizada nos meses de outubro e novembro de 2022, nas bases de dados MEDLINE, *Web of Science*, BDENF e CINAHL e índice bibliográfico LILACS. Não houve delimitação de idioma e de tempo. Os dados foram extraídos e analisados descritivamente por dois pesquisadores independentes. O protocolo de pesquisa foi registrado na *Open Science Framework.*

**Resultados:**

selecionaram-se 26 estudos. As cartilhas, panfletos e folhetos foram as tecnologias mais utilizadas, apresentando os serviços de saúde como o ambiente mais frequente para desenvolver pesquisa sobre prevenção de quedas. As tecnologias desenvolvidas constituíram desfechos importantes: aumento do conhecimento das crianças, dos familiares, cuidadores, profissionais de saúde e de educação.

**Conclusões:**

a utilização de tecnologias educacionais possibilita o aumento de conhecimento, adoção de práticas seguras e redução de quedas.

## RESUMEN

**Objetivos:**

mapear evidencias sobre el uso de tecnologías educativas para la prevención de accidentes por caídas en la infancia.

**Métodos:**

revisión de alcance, realizada en octubre y noviembre de 2022, en las bases de datos MEDLINE, Web of Science, BDENF y CINAHL e índice bibliográfico LILACS. No hubo delimitación de idioma ni de tiempo. Los datos fueron extraídos y analizados descriptivamente por dos investigadores independientes. El protocolo de investigación fue registrado en el Open Science Framework.

**Resultados:**

se seleccionaron 26 estudios. Los cuadernillos, folletos y volantes fueron las tecnologías más utilizadas, presentando los servicios de salud como el ámbito más frecuente para desarrollar investigaciones sobre la prevención de caídas. Las tecnologías desarrolladas fueron resultados importantes: mayor conocimiento de los niños, familiares, cuidadores, profesionales de la salud y la educación.

**Conclusiones:**

el uso de tecnologías educativas posibilita aumentar los conocimientos, adoptar prácticas seguras y reducir las caídas.

## INTRODUCTION

Accident is an unintentional and preventable event that can happen in the home, work, traffic, school, sports and leisure environment, among others, and cause physical and/or emotional injuries^(1)^.

The main causes of accidents with children, registered in the health services, were falls (81%), burns (10%), electric shocks (8.6%), animal bites (7.6%), drowning (6.1%), poisoning (4.9%), crushing (4%), transport accidents (4%), poisoning (1%) and other types of accidents (1.3%). Such accidents occurred 81% of the time at home, 11.3% at another house, 3.9% on the street, 1.9% at school and 2% elsewhere^(2)^. Another survey showed that 52.3% of the accidents happened to children aged zero to five years and 26.2% from six to ten years, with falls (50.7%) were the most common types of accidents in all age groups, confirming that they represent the main reason for injuries in childhood^(3)^.

A fall is the event in which people inadvertently fall to the ground, floor or other level below that on which they were standing^(4)^. Risk factors for children falling are high hammock, presence of stairs or steps without handrails, exits and passages maintained with toys, furniture, boxes or other items that may be obstructive^(5)^, family income, the number of children, the presence or absence of a father, education and lack of spaces for education^(6)^.

Accidents have a significant influence on infant mortality and are responsible for high rates of hospitalization, harming and causing disabilities in child development, including death, thus constituting a problem in public health. It is believed that programmed actions, such as health education activities, focusing on childhood accident prevention, contribute to reducing these occurrences^(7)^. In this regard, there is an imminent need to guide the population on ways to prevent and identify dangerous situations, guaranteeing a safe environment^(8)^.

In this context, educational technologies constitute the central axis of the learning process, as they are resources that enable the mutual construction of knowledge through contextualized education, with the aim of providing opportunities for individuals to take over the role of agents of change^(9)^.

There is a scarcity of review studies on educational technology use for accident prevention due to falls in childhood. Thus, this research can contribute to filling this gap and its results can increase health and education professionals’ knowledge about the educational technologies used for accident prevention due to falls in childhood, in order to guide professional practice in the implementation of interventions that promote safety behaviors in childhood.

## OBJECTIVES

To map evidence on educational technology use for accident prevention due to falls in childhood.

## METHODS

### Ethical aspects

As this is a scoping review, the study was not submitted to the Research Ethics Committee.

### Study design, period and place

This is a scope review, following the review method proposed by the JBI^(10)^, which is a form of knowledge synthesis that addresses an exploratory research question aimed at mapping key concepts, types of evidence and research gaps related to an area by systematically searching, selecting and synthesizing existing knowledge^(11)^. In addition, it proposes recommendations for future research and identifies all relevant literature, regardless of the study design^(12)^.

Synthesis of knowledge is important in health research and practice, as it can make sense of large volumes of primary research^(11)^. The research protocol was registered in the Open Science Framework (https://osf.io/c4z9n).

The search for publications took place in October and November 2022, in the following databases and bibliographic index: Medical Literature Analysis and Retrieval System on-line (MEDLINE via PubMed); Cumulative Index to Nursing and Allied Health Literature (CINAHL); Web of Science; Nursing Database (BDENF - *Base de Dados em Enfermagem*); and in the Bibliographic Index of Latin American and Caribbean Literature in Health Sciences (LILACS - *Literatura Latino-Americana e do Caribe em Ciências da Saúde*). The choice of such databases and bibliographic index is justified by indexing a significant number of studies in health and nursing. It is noteworthy that the list of final references of included studies was analyzed manually, aiming to expand the search and find important studies to be added.

### Inclusion and exclusion criteria

Primary studies that used educational technologies for accident prevention due to falls in childhood were included and the age of less than 18 years was adopted, because it is a literature review in international databases that retrieved publications whose participants were aged 12 years or older, considering them as children^(13, 14, 15, 16, 17, 18)^. There was no delimitation of language or time. Editorials, response letters, theoretical reflection, manuals and those that did not answer the research question were excluded.

Thus, in the bibliographic survey, 2,416 publications indexed in the databases and bibliographic index were identified, to which a publication identified by the list of final references of an included study was added. To prepare the review report, the Preferred Reporting Items for Systematic Reviews and Meta-Analyses Extension for Scoping Reviews (PRISMA-ScR) checklist was adopted^(19)^.

### Study protocol

To conduct the ordination of this scope review, the five steps recommended by the JBI were adopted^(10, 12, 20)^, namely: research question identification; search for relevant studies; study selection; data mapping; grouping, summarizing and presenting the results. The PCC^(10)^ strategy [acronym for Population, Concept and Context] was used to formulate the research question, where: P: infant, preschool and child (childhood); C: educational technologies; and C: accident prevention due to falls. Thus, the following research question was elaborated: what is the evidence on educational technology use for accident prevention due to falls in childhood?

Two reviewers carried out the searches independently and the differences found were discussed and analyzed between them until a final consensus was reached, paying attention to the inclusion and exclusion criteria.

Medical Subject Headings (MeSH) controlled descriptors were used for searches in MEDLINE via PubMed and on the Web of Science, the List of Headings of CINAHL Information Systems, for searches in CINAHL, and Descriptors in Health Sciences (DeCS - *Descritores em Ciências da Saúde*), for searches in BDENF and LILACS via the Virtual Health Library (VHL). Keywords were selected from suggestions of controlled vocabularies and thorough previous reading on the subject. Descriptors and keywords were combined using the Boolean operators OR and AND, according to the particularities of each database and bibliographic index (Chart 1).

**Chart 1:** Final search strategy in each database and the total number of publications retrieved, 2022

Database (Total studies)	Search final strategy
Medline via PubMed (1,909)	((((“child”[MeSH Terms]) OR (“child, preschool”[MeSH Terms])) OR (“infant”[MeSH Terms])) AND ((((((“educational technology”[MeSH Terms]) OR (“health education”[MeSH Terms])) OR (“multimedia”[MeSH Terms])) OR (“communications media”[MeSH Terms])) OR (“audiovisual aids”[MeSH Terms])) OR (“teaching materials”[MeSH Terms]))) AND ((((“accident prevention”[MeSH Terms]) OR (“accidents”[MeSH Terms])) OR (“accidental falls”[MeSH Terms])) OR (“falls”[All Fields]))
CINHAL (295)	((MH “Child”) OR “child” OR (MH “Child, Preschool”) OR “child, preschool” OR (MH “Infant”) OR “infant” ) AND ( (MH “Educational Technology”) OR “educational technology” OR (MH “Health Education”) OR “health education” OR (MH “Multimedia”) OR “multimedia” OR (MH “Communications Media”) OR “communications media” OR “audiovisual aids” OR (MH “Teaching Materials”) OR “teaching materials” ) AND ( “accident prevention” OR (MH “Accidents”) OR “accidents” OR (MH “Accidental Falls”) OR “accidental falls” OR “falls”)
Web of Science (191)	(TS=(child) OR TS=(“child, preschool”) OR TS=(infant)) AND (TS=(“educational technology”) OR TS=(“health education”) OR TS=(multimedia) OR TS=(“Communications Media”) OR TS=(“Audiovisual Aids”) OR TS=(“Teaching Materials”)) AND (TS=(“accident prevention”) OR TS=(accidents) OR TS=(“accidental falls”) OR TS=(falls))
BDENF and LILACS via Virtual Health Library (VHL) (21)	((mh:(*criança*)) OR (mh:(“*pré*-*escolar*”)) OR (mh:(*lactente*))) AND ((mh:(“*tecnologia educacional*”)) OR (mh:(“*educação em saúde*”)) OR (mh:(*multimídia*)) OR (mh:(“*meios de comunicação*”)) OR (mh:(“*recursos audiovisuais*”)) OR (mh:(“*materiais de ensino*”))) AND ((mh:(“*prevenção de acidentes*”)) OR (mh:(*acidentes*)) OR (mh:(“*acidentes por quedas*”)) OR (*quedas*)) AND ( db:(“LILACS” OR “BDENF”))
2,416	-

It should be noted that the descriptor “Adolescent” was not included in the search strategy, because the main focus of the study is infants, and the Child and Adolescent Statute in Brazil considers a child “a person up to the age of twelve incomplete”^(21)^. However, although the descriptors used were strictly related to childhood, searches retrieved some international articles that considered adolescents as children, given that the United Nations Convention on the Rights of the Child understands that “a child is every human being under 18 years of age”^(22)^.

### Data extraction and analysis

Identified publications were exported to the Rayyan^(23)^ application, where duplicates were removed and the title and abstract were analyzed. Next, 35 publications were read in full, including 26 studies. Information was extracted from the items indicated by the JBI^(10)^: identification of authors; year of publication; country of origin; participants; design; main outcomes; educational technology used (intervention); type of accident; and environment (place) where the technology has been implemented.

Evidence was synthesized by two researchers, independently, and disagreements were analyzed until a final consensus was reached. Extracted information was organized for the descriptive synthesis of each of the studies included in this review.

## RESULTS

Database searches retrieved 2,416 articles, of which 25 made up the sample. After reading the selected articles, a study was identified that was included in the final references of an included study and that met the inclusion criteria, being added to the first result, totaling a sample of 26 included articles. A total of 166 publications were removed due to duplicates.

**Figure 1:**
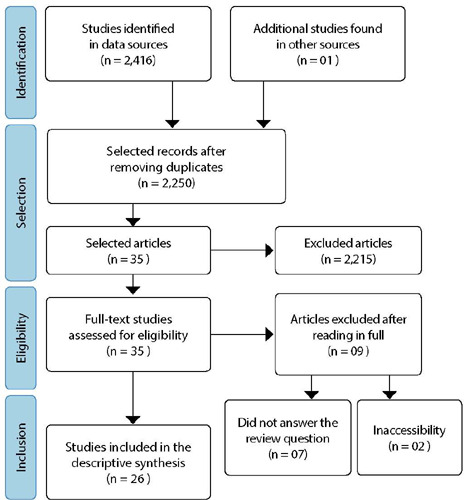
Flowchart according to Preferred Reporting Items for Systematic Reviews and Meta-Analyses Extension for Scoping Reviews (PRISMA-ScR) criteria, 2022

After reading the title and abstract, 2,215 studies were excluded for not answering the research question. Thus, 35 studies were eligible for full reading. Next, nine studies were excluded, seven for not responding to the research question and two for inaccessibility. It is noteworthy that the researchers sent an email to the main author of the two inaccessible studies, requesting articles’ availability, but no response was obtained. The process of identification, selection, eligibility and inclusion of studies is shown in Figure 1.

Chart 2 presents the distribution and synthesis of the included studies according to reference, country, year of publication, educational technology used, type of accident and place of technology implementation.

**Chart 2:** Reference, country, year, type of educational technology used, type of accident and place of technology implementation (n = 26), 2022

Reference/country/year	Educational technology	Type of accident/place
A1^(24)^/Brazil/2013	- Model with the scenario of the “Three Little Pigs” story; adapted, containing a tree, three houses; - A book with the safe version of the story; - Puppets and panel with student responses.	- Bike falls when riding without training wheels; from the tree when climbing to play in the branches; from the window without protection; from the ladder; in the boiling water cauldron/municipal kindergarten school.
A2^(13)^/Canada/2015	- Playground safety checklist; - Method of induced hypocrisy (posters representing safe and risky behavior in the playground); - Photos of children playing on playground equipment (slide, swings, climber, monkey bars) for discussion; - Video clips (DVD); - Signature of the forms “Commitment to avoid risky games on the playground”, “Promise to play safely”; - Role play and discussion guide.	- Falls from local playground/playground equipment and clubs.
A3^(25)^/ New Zealand/1999	- Intervention group: information about the hazards, an engineer’s report, regular contact and encouragement to act on the report, and assistance in obtaining funding; - Control group: information about playground hazards.	- Falls from playground equipment/24 schools.
A4^(26)^/United States/2002	- Computer program for assessment and provision of personalized information entitled “Baby, be safe”; - Customized and generic injury prevention leaflet.	- Falls from stairs and with a walker/primary care pediatric clinic.
A5^(27)^/United States/1977	- Fall report by emergency rooms and police stations, followed by counselling, referral and data collection by public health nurses; - Media campaign to inform the public and raise awareness of the dangers; - Community education for prevention through door-to-door hazard identification, counseling by community workers, community organizing efforts with schools, tenant groups, clinics, churches, health care providers; - Provision of free, easy-to-install window guards for families with young children living in high-risk areas.	- Falls from windows/health centers, hospitals, pediatric clinics, police stations, community councils, children’s health clinics, family health centers, day care centers, career initiation programs, community school councils, child welfare agencies, neighborhood service offices, community corporations, supermarket chains, church pastors and a long list of community-based organizations including neighborhood associations, tenant groups, community service offices, rehabilitation groups housing.
A6^(28)^/Brazil/2015	- Filming of caregiver-baby; - Analysis of caregivers’ behaviors; - Illustrative leaflet and educational video.	- Falls of babies during changing clothes (falls from heights)/two Basic Health Units.
A7^(29)^/Brazil/2009	Educational actions with participatory and transformative pedagogical bases: - The creation and dramatization of the song “*A Lição do Sapeca*”; - The creation of the dynamics of the “Simulated House”; - The creation of the dynamic “What is this?” and dialogue.	- Falls/an early childhood education school.
A8^(30)^/South Korea/2020	- Safe Kids Hospital (SKH) app for children; - Semi-structured interviews, narrated audio and text for caregivers, along with real photos and videos that described the definition and real cases of safety incidents among hospitalized children.	- Falls/an urban medical center.
A9^(31)^/Brazil/2021	- Educational video.	- Fall in a hospitalized child/a university hospital.
A10^(32)^/ South Korea/2021	- Experimental group I: SKH application for children and educational materials for caregivers; - Experimental group II: 31 paper images based on situations of hospital beds, windows, bathrooms, corridors and elevators; - Control group: usual educational intervention for children’s caregivers.	- Falls/pediatric ward of three general hospitals.
A11^(14)^/China/2015	- Intervention group: children, their parents/guardians and the school received a multi-level school-family-individual education, which included a child injury prevention poster in schools, an open letter on safety instruction for parents/guardians and education in health in various media (Microsoft PowerPoint lectures, videos, manuals) for children; - Control group: children received manual education only.	- Falls/two primary schools and three secondary schools.
A12^(33)^/Singapore/2013	- A multilingual poster on fall prevention has been strategically placed next to all cribs.	- Falls/a pediatric acute care hospital.
A13^(15)^/United States/2014	Fall prevention program: - Educational poster and the Humpty Dumpty Falls Scale for nurses; - Educational booklet for parents: “Preventing Falls, Increasing Safety”.	- Falls in the hospital/pediatric unit of a medical center.
A14^(34)^/France/2003	- Group 1: counseling, two pamphlets on domestic injuries and prevention methods, emergency telephone numbers and a safety kit. - Group 2: counseling and pamphlets, but did not receive the kit.	- Falls in the bathtub, stairs, walker due to slipping on rugs, carpets and wires, from high chairs, balcony and window/home visits.
A15^(35)^/United States/2018	- A parental security agreement; - Education, re-education and modeling of safe sleep practices; - Implementation of a reproting and debriefing system for falls in children.	- Newborn falls in the hospital and at home/Intensive Care Unit of a hospital.
A16^(16)^/Canada/2008	- Construction of posters; - Intervention of hypocrisy (review the posters and interview); - Interview with questionnaires.	- Falls on playground/school equipment.
A17^(7)^/Brazil/2021	Educational game prototype for common childhood accident prevention.	- Fall from bed and in the playground/scenarios: home, public road, school environment and public square or park.
A18^(36)^/Canada/2013	- Videos addressing the theme “safe children” and a post-video discussion; - Parents received active supervision for a month to assess adopted safety practices.	- Falls/home.
A19^(37)^/England/2019	- Grow up Safely (GUS) Android mobile app, designed to enhance parents’ knowledge and understanding of potential injury risk areas.	- Furniture falls/service at a local children’s center.
A20^(38)^/England/2007	- Educational program Risk Watch Injury Prevention, using risk watch folders and ‘‘risk boxes’’ as teaching resources.	- Falls from stairs/primary schools.
A21^(17)^/United Kingdom/2006	- Injury prevention program: the Citizen Safety Project (CSP).	- Falls/rural secondary school and primary school.
A22^(18)^/Sweden/2012	- Motala program implementation to prevent serious and less serious unintentional child injuries.	- Falls at home, schools and sports/community facilities.
A23^(39)^/Taiwan/2009	- Warning posters about accidental falls at the bedside; - Education related to the family through educational cards about falls; - Marking of the nursing care plan to remind nurse caregivers to comply with accidental falls education; - Highlight families whose children may belong to the high-risk group and carefully monitor these patients; - Monthly verification of the safety of the bedside railings	- Fall in hospital bed/pediatric observation unit.
A24^(40)^/United States/2020	- Sessions.	- Falls/semi-urban clinic of the Special Supplemental Nutrition Program for Women, Infants and Children and an urban pediatric primary care clinic.
A25^(41)^/Canada/2009	- Educational video.	- Falls/home.
A26^(42)^/Canada/2014	- The Million Messages is an innovative program that partially uses print media to provide families with childhood injury prevention messages.	- Fall at home (bathtub, stairs and toilet)/local community.

The results showed articles published between 1977 and 2021, more frequently in 2009, 2013, 2015 and 2021 (n=03; 11.5%, each). Studies conducted in Brazil (n=05; 19.2%), Canada (n=05; 19.2%) and the United States (n=05; 19.2%) predominated. Among the educational technologies analyzed, those that used booklets, pamphlets and leaflets (n=14; 53.8%), counseling and guidance (n=09; 34.6%), video clips (n=07; 26, 9%), forms and questionnaires (n=07; 26.9%) and computer programs and applications (n=05; 19.2%) were identified. It is important to emphasize that some publications used more than one technology. Regarding the types of falls, falls in general (n=09; 34.6%) stood out with a higher frequency of studies carried out in health services (n=11; 42.3%).

Chart 3 presents the descriptive summaries of articles included in the review. As for study design, there was a higher frequency of randomized controlled studies (n=10; 38.5%) carried out with children under 15 years old (n=12; 46.2%). Regarding the outcome, the studies highlighted the increase in knowledge of family members, caregivers and professionals (n=09; 34.6%) and the change in behavior to prevent falls (n=07; 26.9%).

**Chart 3:** Descriptive synthesis of articles included in the review (n = 26), 2022

Article	Design/Participants	Outcomes
A1^(24)^	Descriptive study with 30 students (five and six years old) and a teacher.	The activities aroused children’s attention about risk situations for accidents and prevention strategies. The educational material was well prepared and can be extended to the whole school.
A2^(13)^	Experimental study with children aged 7 to 12 years/experimental group - 80 children; control group - 24 children.	The program was easy to implement and effective in positively changing injury beliefs about children’s risky practices on playground equipment.
A3^(25)^	Clinical trial with 24 schools.	There was a significant drop in risk in intervention schools compared to control schools. Intensive intervention is more effective than just providing information.
A4^(26)^	Randomized controlled study with 174 parents of children aged between 6 and 20 months.	Personalized communications were found to be more effective in promoting injury prevention behaviors than generic print media.
A5^(27)^	Two-year pilot program that was developed by combining service with research.	There was a significant reduction in falls. The program is a solution to an urgent urban problem that other cities might consider to avoid loss of life and limb, and the corollary financial, burden for hospitalization, rehabilitation and maintenance of the injured and permanently disabled.
A6^(28)^	Descriptive and exploratory research with 25 caregiver-infant dyads.	Observing babies’ and their caregivers’ behavior is essential to identify risk situations for falls from the most varied places and heights. The video seems to be a good strategy for promoting safety behaviors and preventing accidental falls in babies.
A7^(29)^	Qualitative research with 11 professionals, 18 children and 20 family members.	The health team set in motion the process of community mobilization to prevent childhood domestic accidents. Communication was effectively established between the researcher and the health team and between the team and the children and parents. The playfulness of educational technologies favored subjects’ acceptance and their experiences.
A8^(30)^	Quasi-experimental study with 30 hospitalized preschool children and 30 caregivers.	The level of security awareness increased after the security incident prevention program using the SKH application. Participants found the app easy to use and a fun way to learn. Children showed high levels of satisfaction with children’s characters and high levels of curiosity, and they enjoyed continuing to play.
A9^(31)^	Methodological research with 13 expert judges and nine health professionals.	The educational video prepared, “Fall prevention in hospitalized children”, proved to be valid in terms of content and appearance by both the judges and the target audience, with the potential to mediate educational practices in a hospital context as well as training health professionals in child health care.
A10^(32)^	Randomized controlled clinical trial with 116 hospitalized children aged between 3 and 6 years and their caregivers.	Hospital safety awareness increased more after the intervention in experimental groups I and II than in the control group. It is considered a useful educational intervention to prevent safety incidents in clinical areas.
A11^(14)^	Experimental research with children from 08 to 16 years old.	The multilevel education intervention can significantly increase knowledge and attitude scores for accidental injuries, as after the intervention, injury incidence decreased. It should help children change their risky behaviors and reduce accidental injury incidence.
A12^(33)^	Quasi-experimental study with 30 children aged 3 years or less.	The presence of a fall prevention poster to remind parents/caregivers to safely lift and lock crib rails at all times was effective in reducing the number of falls.
A13^(15)^	Quality improvement project – pilot study with 29 pediatric nurses.	After reading and reviewing the educational poster, all 29 nurses were able to correctly answer the 8-question drop post-test. For nurses, the Humpty Dumpty Falls Scale was useful for their clinical practice, increasing their awareness of patients at risk of falling, and helping them to implement safety precautions.
A14^(34)^	Randomized study, with two groups of 50 people. Families of children aged at least 6–9 months.	Focus group meetings with health professionals were relevant both for the process assessment and for the impact. Home delivery of the kit was considered conducive to discussion and the effectiveness of the intervention, raising awareness of potential household hazards and leading to reflection on safety. The questionnaire was also perceived positively and was considered a vehicle for providing information.
A15^(35)^	Observational and descriptive study with nurses and parents.	A newborn safety package was implemented to promote safe sleep and minimize the risk of falls in the acute care setting. Parents and nurses working together create a safe environment to protect patients from harm.
A16^(16)^	Case-control study with 239 children aged 2 to 13 years.	The hypocrisy-induced intervention was effective in reducing children’s risk-taking intentions. Most children who received the intervention no longer intended to engage in risky behaviors that they had previously endorsed.
A17^(7)^	Descriptive study.	The prototype is a potential playful instrument to be implemented to mediate child health education actions.
A18^(36)^	Randomized clinical trial with 186 mothers.	After the intervention, parents showed a decrease in the time their child was completely unsupervised, an increase in the time they kept their child in sight, and an increase in the level of supervision provided when the child was out of sight.
A19^(37)^	Randomized clinical trial with parents/caregivers of infants who are not yet crawling, parents aged 23 years or younger, and parent-infant group.	Most participants felt that the information included in the app was well written and informative and had the right level of information. Two of the groups commented that the information was succinct and easy to read, with one participant stating that it was very well written, easy to read and interesting.
A20^(38)^	Cluster-randomized controlled trial, carried out in 20 schools and with 459 children.	The first year of a teacher-led educational program in primary schools was effective in increasing some aspects of children’s safety knowledge and skills.
A21^(17)^	Controlled study in secondary school (22 students) and rural secondary school (55 students).	The idea of peer mentoring of an accident prevention and risk awareness project was perceived very positively, as was the transferable nature of the Citizen Security Project for other years.
A22^(18)^	Quasi-experimental design.	The program was only partially successful in that it reduced the injury rate in employed households, but did not influence the injury rate in self-employed households for boys and non-professionally active households.
A23^(39)^	Prospective study with pediatric patients admitted to the pediatric observation unit.	The occurrence of accidental falls decreased significantly in the third period. After performing the last intervention, an improvement of 100% was achieved.
A24^(40)^	Randomized study with 277 mother-infant dyads.	Among families with multiple home security issues, the intervention resulted in relatively large effects, whereas the effects for families with no/few home security issues were small and not significant.
A25^(41)^	Descriptive study with mothers of young children.	The results provide information on strategies that promote commitment to more closely supervise mothers as well as strategies to prevent accidents. Future research will need to determine whether the findings generalize to parents or other caregivers.
A26^(42)^	Descriptive study with 60 mothers of young children.	Portraying the consequences of injuries and displaying appropriate negative emotions on children’s face would affect parents’ perceptions of safety messages in addition to the effects achieved by describing the risk of injuries. Results confirmed that these image characteristics have an important effect on parents’ attention to the message and risk assessment.

Regarding the results of selected studies, four categories were identified according to the place of implementation: *Technologies applied in schools*; *Technologies applied in the community*; *Technologies applied at home*; and *Technologies applied in health services*. The categories and technologies used are presented in Chart 4. It is noteworthy that some studies developed research in different environments, being inserted in more than one category.

**Chart 4:** Categories according to the place of implementation and the technologies used (n = 26), 2022

Categories	Technology
Technologies applied in schoo ls^(14, 16, 17, 24, 25, 27, 29, 38)^	Booklets, pamphlets and leaflets; video clips; dynamics; guidelines and advice; forms and questionnaires.
Technologies applied in the community^(13, 18, 27, 37, 42)^	Booklets, pamphlets and leaflets; video clips; guidelines and advice; computer programs, applications and games; Forms and questionnaires; Media campaigns.
Technologies applied at home^(7, 27, 34, 36, 41)^	Booklets, pamphlets and leaflets; guidelines and advice; computer programs, applications and games; video clips; provision of safety equipment.
Technologies applied in health services^(15, 26, 27, 28, 30, 31, 32, 33, 35, 39, 40)^	Booklets, pamphlets and leaflets; video clips; guidelines and advice; computer programs, applications and games; forms and questionnaires; scale.

## DISCUSSION

The recognition of the fall as a safety incident, which can generate temporary and permanent disabilities, encouraged the development of technologies that seek measures, such as prevention strategies and increased awareness of caregivers as well as the early identification of risks^(42, 43)^.

In addition to identifying the factors involved in the occurrence of accidents, there is still a need to implement proposals that enable the applicability of different forms of promotion and childhood domestic accident prevention, including falls^(29)^. In this study, there was evidence of an increase in the interest of researchers in the search for the construction of practices that aim to prevent falls in childhood, and it is possible to observe a variety of technologies constructed in different environments^(7, 13, 14, 15, 16, 17, 18, 24, 25, 26, 27, 28, 29, 30, 31, 32, 33, 34, 35, 36, 37, 38, 39, 40, 41, 42)^.

In this way, technologies were identified that prioritized fall prevention through booklets, pamphlets and leaflets, video clips, guidelines and advice, computer programs, applications and games, forms and questionnaires, media campaigns, among others.

Furthermore, the diversity of research environments was observed, such as schools, the community, the home and health services, subsidizing professional and caregiver practices, leading to the planning, construction and execution of different approaches^(7, 13, 14, 15, 16, 17, 18, 24, 25, 26, 27, 28, 29, 30, 31, 32, 33, 34, 35, 36, 37, 38, 39, 40, 41, 42)^.

Considered as a privileged environment for carrying out preventive and health-promoting activities, schools are not exempt from the risk of falls and other accidents, especially during games^(24)^. In this regard, education in schools is an important tool to work on fall prevention, since the implementation of educational strategies in parks and other areas outside school makes it possible to identify risks with children and reflect on possible changes in the environment and behavior^(17, 24)^.

Among the technologies used in the school environment are booklets, pamphlets and leaflets. It is noteworthy that using validated booklets as educational practices has become essential for achieving results applied in different environments and with diverse populations^(44)^. Using written approaches increases the perception that the result is closer to reality, and using images and symbols increases text understanding, conveying safety information to a diverse audience. Moreover, the presentation of information through written technologies makes it possible to reach audiences from different social strata, since it is a more accessible intervention^(42)^.

Educational technologies allow access to other intelligences and skills, inserting written and unwritten language, which results in a greater approximation of users and professionals with the theme presented^(44)^. Educational technologies’ playfulness had a strong impact on the participation and interaction of children, parents and professionals during research, which enabled effective communication between researchers and participants^(27, 29)^. In addition to this, it is important to emphasize the need for interaction between teachers and health professionals, as using educational strategies that can be developed in schools allows the identification of risks and encourages reflection on adaptations in the environment and behavior^(14, 24, 25)^.

It is important to highlight the need to implement educational measures in the community in order to prevent injuries in children, seeking to include the entire population as an agent for maintaining infants’ health, since they also frequently suffer injuries in environments outside the home^(13)^.

The technologies implemented in the community seek the participation of all, with the purpose of providing effective actions, changes in the scenario and democratization of knowledge, collaboratively developing strategies that promote children’s health and fall prevention^(45)^.

It should be noted that cultural or environmental factors can be attributed as the primary causes of falls in children^(27)^. Play environments, such as playgrounds, have been identified as a place that poses a risk of falling for children, especially of school age, sometimes producing serious injuries that require medical treatment^(13)^.

Among the technologies applied in the community, the implementation of different programs stood out, such as safety checklists, reporting of falls followed by counseling and referrals, home security supervision programs and mobile application development, described as promising methods that achieved important results in participants’ attention and risk assessment^(13, 18, 27, 37, 42)^.

Developing educational interventions in the community requires standardization and a rigorous approach during its applicability in order to obtain effectiveness. However, its applicability is of great relevance in injury prevention, be it accident or illness prevention in childhood, being considered a valid tool in the implementation of educational actions in health and as an intermediary of information to preserve health, promoting the circulation of accurate information to the entire community^(13,46)^.

The home environment is a place with frequent occurrences of falls involving children, influenced by the physical structure and organization or arrangement of objects or furniture. Additionally, investigations indicate that there is a direct relationship between the economic profile of families living in situations of social vulnerability and domestic accidents. Moreover, the lack of resources makes it impossible to access protective structures that contribute to fall reduction in children^(5, 27, 34)^.

For this, the adoption of educational measures becomes increasingly necessary, seeking to reduce accidents and, consequently, prevent injuries that generate psychological trauma and irreparable sequels^(5)^. Among the technologies used at home, the highlights were booklets, pamphlets and leaflets as well as guidelines and advice, computer programs, applications and games, video clips, and provision of safety equipment^(7, 27, 34, 36,, 41)^.

Education strategies with posters, guidance to family members by health professionals, regular checking of environmental conditions and scale use are educational technologies used to train professionals and to help identify children at risk of falling^(47)^.

The insertion of educational technologies produces a significant change in the behavior and vision of parents and other guardians, as it encourages the adoption of safety measures at home, an increase in the level of supervision of children, greater attention to children, generating changes in supervision practice and illness prevention^(36)^.

It is noteworthy that the period of home accidents occurs mainly at the beginning of development, when children do things that parents do not expect and are not prepared for. In this way, it is important to provide educational messages to parents to facilitate the process of commitment and behavioral changes that allow them to supervise more closely and, at the same time, promote children’s independence to perform tasks at home^(41)^.

In addition, a challenge to be overcome is to increase male participation in community programs regarding child care, as it may contribute to expanding the family value related to home security conditions^(29)^.

Falls are the most common incidents related to safety in hospitalized children^(48)^, and risk factors for falls in hospitalized children include not using bedside rails, child restlessness, broken beds and lack of time for nurses to explain in detail the need for fall prevention^(40)^. It is noteworthy that unintentional falls of newborns in the hospital environment cause injuries such as edema, hyperemia in the temple and knee, parietal bone fracture and hematoma^(49)^.

Among the technologies used in the hospital context, the Humpty Dumpty Falls Scale, applied by nurses, stands out, a screening resource for assessing the risk of pediatric falls in inpatient and outpatient settings, associated with the educational leaflet entitled “Preventing falls, increasing safety”, which explains the staff’s concerns and outlines what parents and children should do and what to avoid to ensure pediatric patient safety. The leaflet was added to the pediatric unit admission packet and given to each family^(15)^.

The videos were also used as an educational action in primary care services with caregiver-baby^(28)^ and hospital services with health professionals^(31)^. The visual information was the one that most caught the attention of the participants, especially the images that showed risk situations for falls when changing babies’ clothes. The video, in turn, can collaborate to avoid childhood accidents and enable changes in behavior^(28)^ as well as mediate educational practices in the hospital context^(31)^.

Moreover, mobile applications were delivered to hospitalized children and their caregivers, which enabled an increase in knowledge and a change in safety behaviors, as they are easy to use and promote learning^(30, 32)^. The feasibility, acceptability and effectiveness of SKH among preschoolers, which constitutes a useful educational method to raise awareness about hospital safety incidents, as using animated materials has proven to be effective for early childhood education^(30)^.

Health professionals can intervene on the hospital environmental factors that contribute to the fall and guide family members regarding the necessary care^(31)^. It was found that among the places where educational technologies were implemented, only four studies were carried out in primary care services^(26, 27, 28, 40)^, which indicates the need to expand educational activities during prenatal consultations, childcare and home visits.

### Study limitations

Evidence analysis was restricted to the studies retrieved from the aforementioned databases and bibliographic index. So, there is the possibility that the applied search strategies did not find other publications relevant to the research. Another limitation is the fact that some studies have worked with the prevention of various accidents, including falls, however, without discriminating which types of falls. Furthermore, the studies’ scientific rigor was not assessed.

### Contributions to health

The evidence identified in this review presents as a contribution the construction of knowledge about the different types of educational technologies existing in the literature for fall prevention in children, applied in different contexts. The present study presents valid educational alternatives capable of subsidizing health and education professionals to expand their knowledge and take ownership of such resources, with the aim of implementing activities that promote safety behaviors.

## CONCLUSIONS

The concern for children’s safety has enabled the development and application of various educational technologies for preventing different types of falls in school, hospital, home and community contexts, incorporating the participation of infants, parents, caregivers and health and education professionals, showing that it is a responsibility of the whole society.

Using educational technologies, in turn, caught children’s attention. There was a decrease in risk of accidents, safe behavior promotion, fall reduction, increase in knowledge and attitudes for accidental injury prevention, increasing the level of parental supervision, among others.

Finally, the failure to identify the types of falls that were addressed in some articles, the absence or incipient participation of a father in educational activities and little research implemented in primary care services emphasize the need to carry out studies that broadly include the father figure in the processes of identifying risk factors, dangerous situations and ways of preventing falls, with primary care spaces as privileged places for health education interventions.
